# Computational Seebeck Coefficient Measurement Simulations

**DOI:** 10.6028/jres.117.009

**Published:** 2012-05-02

**Authors:** Joshua Martin

**Affiliations:** Material Measurement Laboratory, National Institute of Standards and Technology, Gaithersburg, MD 20899

**Keywords:** Finite element analysis, Seebeck coefficient, thermoelectric

## Abstract

We have employed finite element analysis to develop computational Seebeck coefficient metrology simulations. This approach enables a unique exploration of multiple probe arrangements and measurement techniques within the same temporal domain. To demonstrate the usefulness of this approach, we have performed these Seebeck coefficient measurement simulations to quantitatively explore perturbations to voltage and temperature correspondence, by comparing simultaneous and staggered data acquisition techniques under the quasi-steady-state condition. The results indicate significant distortions to the Seebeck coefficient and a strong dependence on the time delay, the acquisition sequence, and the probe arrangement.

## 1. Introduction

Thermoelectric effects enable the interconversion of thermal and electrical energy. This conversion process is governed by two primary phenomena: the Seebeck effect and the Peltier effect [1–7]. The Seebeck effect describes the proportional electric potential emergent across a conductor in a uniformly applied thermal gradient. The value of this ratio is termed the Seebeck coefficient:

(1) $$S_{ab}=\underset{\Delta T\to 0}{\lim}\frac{\Delta V_{ab}}{\Delta T},$$

where Δ*V_ab_* is the electric potential between two materials *a* and *b*, and Δ*T* is the temperature difference. In the Peliter effect, the transmission of an electrical current through the interface of two dissimilar conductors results in the liberation or the absorption of thermal energy at the interface. These effects are the physical mechanisms for power generation and solid-state refrigeration in thermoelectric devices, respectively.

The Seebeck coefficient is a physical property that singularly identifies a material’s potential thermoelectric performance. Measurement of this parameter is essentially a practice in low voltage measurements requiring careful attention to the electrical and thermal contact interfaces [8–13]. In most implementations, as few as three voltage measurements are required: one for the generated thermoelectric voltage Δ*V* and one each for the hot and cold thermocouple voltages that determine *T*_2_ and *T*_1_, respectively. Furthermore, it is essential that the electric potential and temperature difference be acquired at the same location and at the same time. The various methods are best defined by the behavior of base sample temperature and by the gradient heating technique. In the differential method, a small thermal gradient is applied to the sample at an average temperature of interest *T_o_*. The Seebeck coefficient can then be obtained by the ratio of the electric potential and the temperature difference according to Eq. (1), provided Δ*T/T_o_<<1*, and Δ*S_ab_/S_ab_<<1*, when *V_ab_* ∝ *T_o_*.

By describing the behavior of the thermal gradient, differential methods can be further categorized into three conditions: steady-state (DC), quasi-steady-state (qDC), and transient (AC), assuming the *observation* time scale, i.e., the time interval required to measure one voltage channel [8]. This is usually larger than the aperture time of the voltmeter, defined as the integration time of the analog to digital converter. Under steady-state conditions, the Seebeck coefficient is often calculated from the linear fit of multiple electric potential/temperature difference data points rather than one to avoid the assumption that the experimental data are collinear with the ordinate (*V_ab_* = 0, Δ*T* = 0). This can effectively eliminate any offset voltage. A more rapid approach uses a continuously increasing heat flux rather than multiple steady-state Δ*T*’s. However, many implementations of this qDC technique incorporate voltage channel switchers and therefore a staggered acquisition. During the finite time required to switch and measure the next voltage channel there will likely be sufficient thermal drift to distort the temperature-voltage correspondence, essentially ‘smearing’ the Seebeck coefficient. To fully characterize these distortions experimentally suggests an impractical number of sample contacts and voltage meters.

The software package COMSOL Multiphysics^1^ has been used previously to successfully model thermoelectric Peltier and power generation module device performance [14–16]. We have therefore employed a similar finite element analysis approach to develop computational Seebeck coefficient metrology simulations. This approach enables a unique exploration of multiple probe arrangements and measurement methodologies within the same temporal domain under ideal conditions. To demonstrate the usefulness of this approach, we have performed Seebeck coefficient measurement simulations comparing simultaneous and staggered data acquisition techniques under the quasi-steady-state condition. These results quantify the errors arising from distortion of the temperature-voltage correspondence. In addition, the results lead to some insightful recommendations to reduce measurement uncertainty.

## 2. Simulation Model

COMSOL Multiphysics provides a finite element modeling and simulation environment for solving systems of partial differential equations (PDEs). These stationary or time-dependent equations may be solved for multiple coupled multiphysics phenomena. For a thermoelectric analysis, the thermal transfer and continuity of electric charge equations can be simultaneously coupled by the thermoelectric constitutive equations [17] and the constitutive equation for a dielectric medium to produce a system of coupled thermoelectric field equations [14,16]:

(2) $$-\vec{\nabla }\left(\left(\sigma S^{2}T+\lambda \right)\vec{\nabla }T\right)-\vec{\nabla }\left(\sigma ST\vec{\nabla }V\right)=\sigma \left({\left(\vec{\nabla }V\right)}^{2}+S\vec{\nabla }T\vec{\nabla }V\right)$$

(3) $$\vec{\nabla }\left(\sigma S\vec{\nabla }T\right)+\vec{\nabla }\left(\sigma \vec{\nabla }V\right)=0,$$

where *σ* is the electrical conductivity, *S* is the Seebeck coefficient, *λ* is the thermal conductivity, and *V* and *T* are the field variables for voltage and temperature, respectively. These equations must first be transformed into COMSOL’s coefficient form for the arbitrary PDE-application mode, defined as follows:

(4) $$C_{a}\frac{\partial^{2}u}{\partial t^{2}}+d_{a}\frac{\partial u}{\partial t}+\nabla \cdot \left(-c\nabla u-\alpha u+\gamma \right)+\beta \cdot \nabla u+au=f,$$

in a region Ω, where

(5) $$n\cdot \left(-c\nabla u-\alpha u+\gamma \right)+qu=g-h^{T}u\;\text{on}\;\partial \Omega$$

(6) hu=ron∂Ω

define the coefficients for the Neumann and Dirichlet boundary conditions, respectively, on the surface of region Ω. For the vector field variable

(7) $$\vec{u}=\left(\begin{array}{c} \begin{array}{c} T\\ V \end{array} \end{array}\right),$$

the resulting coefficients for the thermoelectric field equations in this PDE form are [15,16]:

(8) $$c=\left(\begin{array}{cc} \lambda +\sigma S^{2}T & \sigma ST\\ \sigma S & \sigma \end{array}\right),$$

(9) $$f=\left(\begin{array}{c} \sigma \left({\left(\vec{\nabla }V\right)}^{2}+S\vec{\nabla }T\vec{\nabla }V\right)\\ 0 \end{array}\right),$$

and

(10) $$d=\left(\begin{array}{c} \rho C\\ 0 \end{array}\right),$$

where *ρ* is the density and *C* is the heat capacity. The coefficient *d* is zero for static conditions but for transient conditions incorporates the displacement current associated with capacitive influences [14]. However, this component is a minor contributor for slower transient processes. All other unspecified coefficients are zero.

## 3. Simulation Methodology and Results

The geometry model comprises a 14 mm × 2.5 mm × 2.5 mm n-type Si_80_Ge_20_ semiconducting thermoelectric element contacting two 2.5 mm × 2.5 mm × 2.5 mm tungsten probes on each end. Isotropic material property data were then assigned to each of these subdomains for tungsten [18] and for the Si_80_Ge_20_ element [19]. The temperature dependent transport property data (*σ*, *S*, and *λ*) for n-type Si_80_Ge_20_ were extracted from [20] and interpolated by cubic splines within COMSOL. These material parameters provide a reference data set with which to compare the simulated Seebeck coefficient results.

**Fig 1:**
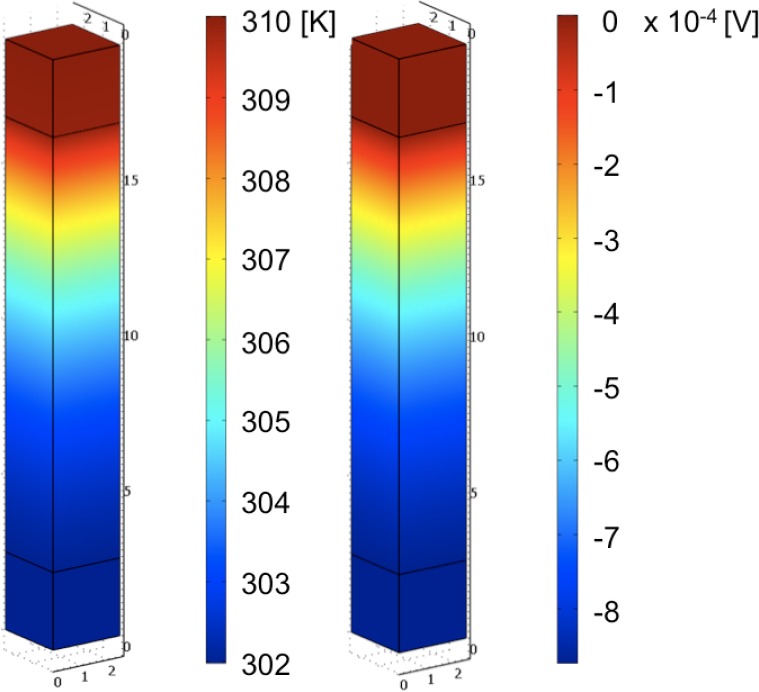
Temperature (left, in K) and voltage (right, in V) distribution on the thermoelectric model geometry at *t* = 20 s.

For simplicity, these simulations assume continuous boundary interfaces between the probes and the semiconducting thermoelectric element. Any possible chemical reactions between these materials and within each material are also ignored. To impose a simulated thermal gradient under the qDC condition, the top tungsten probe surface is set to an appropriate boundary condition with a time dependent temperature rise (0.5 K/s). Therefore, after 20 s the maximal temperature difference across the thermoelectric segment will be ≈0.03*T*. All remaining surface boundary conditions are adiabatic, assuming negligible convective and radiative thermal losses to the environment and between the probes and the thermoelectric element. Figure 1 illustrates the thermal gradient and generated voltage gradient on this model geometry at 20.0 s near 300 K.

**Fig. 2:**
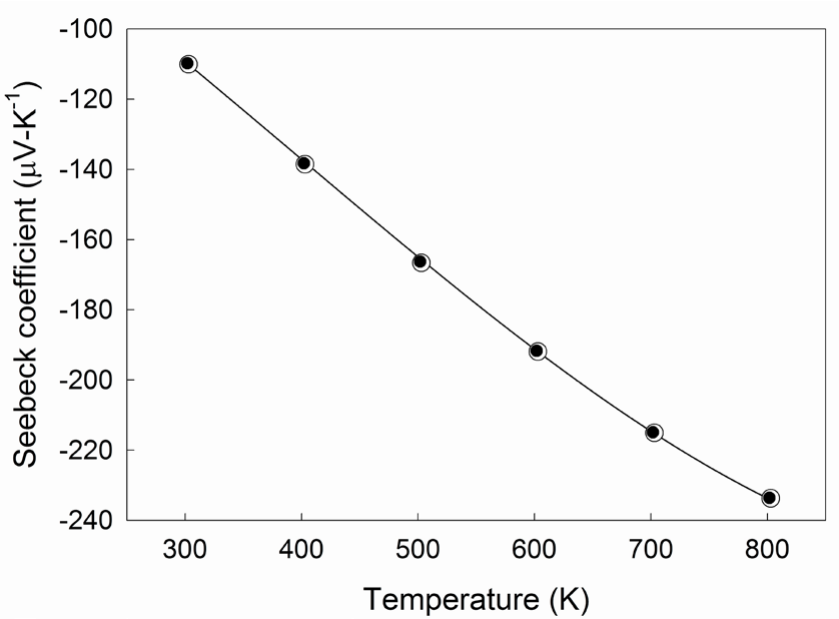
Temperature dependence of the Seebeck coefficient as calculated from the simulated results for the 2-probe (○) and 4-probe (•) arrangement, in comparison to the cubic spline fit of the experimental data.

A time dependent solver was implemented under the following protocols. For each selected temperature (300 K to 800 K in 100 K increments), the simulated *V* and *T* results were recorded as a function of time (0.1 s increment) and position. In the 2-probe arrangement, the temperature difference and the electric potential are commonly measured on the probes which are in direct contact with the ends of the sample [8]. However, many Seebeck coefficient apparatus also sequentially measure resistivity, requiring voltage contacts spatially separated from the ends of the sample. In the 4-probe arrangement, the temperature difference and the voltage difference are measured at two points on the sample equidistant from the hot and cold sinks. Measurement locations were therefore selected at the top and bottom end of the thermoelectric segment to model the 2-probe arrangement and at positions 4 mm apart, vertically centered on the thermoelectric segment to model the 4-probe arrangement. A 4 mm probe spacing represents an appropriate spacing in this geometry as required by resistivity measurements [8]. The Seebeck coefficient was then calculated for both arrangements from the linear fit of multiple electric potential/temperature difference data points, at 2 s, 5 s, 10 s, 15 s, and 20 s, assuming simultaneous acquisition. The results for both arrangements are plotted in Fig. 2. These data are in agreement with the Seebeck coefficient and residual (≈0.4%) as calculated from the cubic spline fit of the experimental data.

The calculated Seebeck coefficients obtained for the 2- and 4-probe arrangement are similar, with *S* = −110.1 *µ*V/K and *S* = −109.8 *µ*V/K, respectively at 300 K. The trace differences between the two arrangements are due to the slightly lower average sample temperature for the 4-probe arrangement and are a reasonable observation from a nonlinear temperature distribution. Since these simulations assumed continuous interface conditions, they do not demonstrate any discrepancy in the Seebeck coefficient between the two probe arrangements throughout the simulated temperature range, as these errors would depend on the thermal/electrical interface between the temperature probes and the thermoelectric segment.

To quantitatively explore perturbations to the *V* and *T* correspondence, additional Seebeck coefficients were calculated by combining *V* and *T* data representing a 0.1 s, 0.5 s, and 1.0 s time delay between each acquisition. These time delays were selected based on standard commercial instrumentation. The Seebeck coefficients were then obtained from the linear fit of multiple electric potential/temperature difference data points beginning at 2 s, 5 s, 10 s, 15 s, and 20 s. However, the Δ*V*, *T*_2_, and *T*_1_ acquisition sequence will further influence the correspondence distortion. Only the four most experimentally reasonable sequences were explored: those pairing the two temperature measurements. Table 1 compares the time delayed Seebeck coefficients calculated for the two sequences that exhibit the greatest distortion, *V*:*T*_2_:*T*_1_ and *T*_1_:*T*_2_:*V*, for the 2- and 4-probe arrangement at 300 K. These data are compared to results obtained from simultaneous acquisition (Δ*t* = 0), indicating a change in the absolute Seebeck coefficient up to ≈8% for the 2-probe and up to ≈17% for the 4-probe arrangement. Combining and averaging each sequence/time delay with its corresponding inverse yields more consistent Seebeck coefficient values that are closer to the expected value (at Δ*t* = 0).

**Fig. 3:**
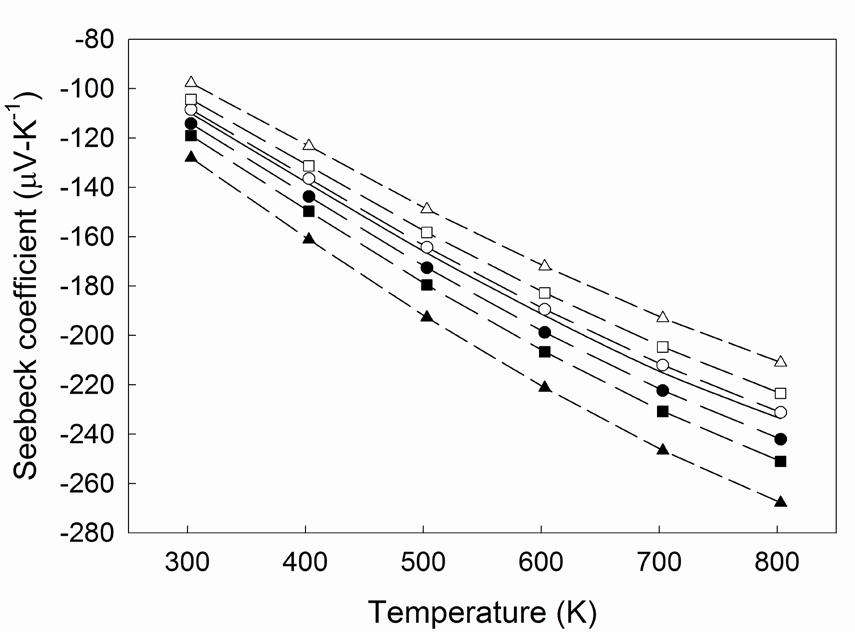
Temperature dependence of the Seebeck coefficient as calculated from the simulated results for the 4-probe arrangement representing a delay of 0.2 s (circles), 0.5 s (squares), and 1.0 s (triangles) for the sequence *V*:*T*_2_:*T*_1_ (filled symbols) and *T*_1_:*T*_2_:*V* (unfilled symbols). The solid line is the cubic spline fit of the experimental data. Dashed lines are a guide for the eye.

Figure 3 shows the temperature dependence of the Seebeck coefficient for the 4-probe arrangement, representing a time delay of 0.2 s, 0.5 s, and 1.0 s for the sequence *V*:*T*_2_:*T*_1_ and *T*_1_:*T*_2_:*V*. The trends suggest that measuring *T*_2_ before *T*_1_ artificially reduces the temperature difference, thereby overestimating the absolute Seebeck coefficient. Correspondingly, measuring *T*_1_ before *T*_2_ underestimates the absolute Seebeck coefficient. These temperature dependent data further illustrate the error that staggered acquisition can introduce in the Seebeck coefficient.

**Fig. 4:**
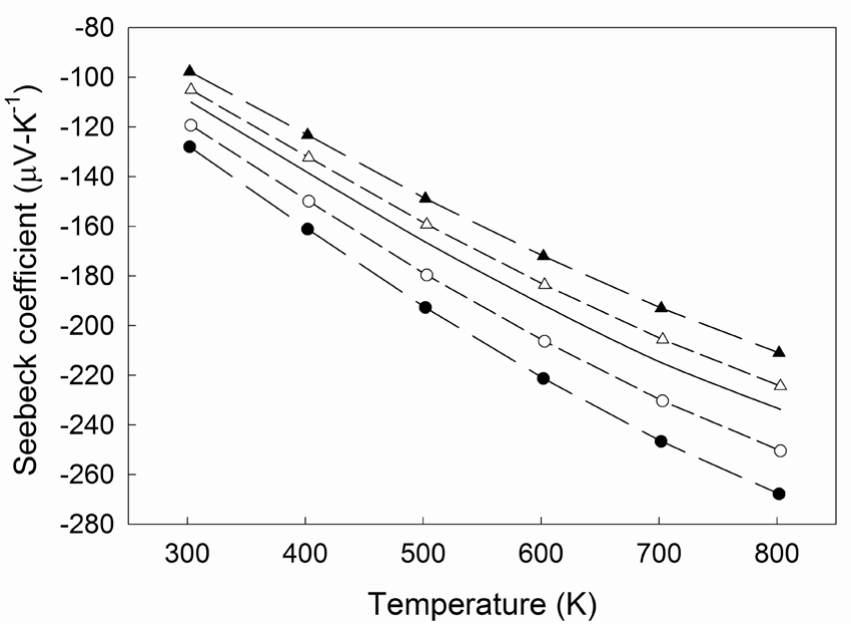
Temperature dependence of the Seebeck coefficient as calculated from the simulated results for the 2-probe (unfilled symbols) and the 4-probe (filled symbols) arrangement representing a delay of 1.0 s for the sequence *V*:*T*_2_:*T*_1_ (circles) and *T*_1_:*T*_2_:*V* (triangles). The solid line is the cubic spline fit of the experimental data. Dashed lines are a guide for the eye.

Figure 4 plots the temperature dependence of the Seebeck coefficient as calculated from the simulated results comparing the 2- probe and the 4-probe arrangement, representing a delay of 1.0 s for the sequence *V*:*T*_2_:*T*_1_ and *T*_1_:*T*_2_:*V*. These conditions represent the greatest distortion to the *V* and *T* correspondence. The 4-probe arrangement clearly demonstrates a greater sensitivity to the time delay. This is reasonably due to the faster rate of change in *V* and *T* nearer the center of the thermoelectric segment, as compared to the ends of the sample, which are in closer proximity to the thermal source and sink. Therefore, the qDC condition introduces less error for the 2-probe arrangement under similar heating rates and under the stated assumptions. As noted previously, these results are simulated under ideal contact conditions that may not fully reflect the experimental comparison of both probe arrangements.

To mitigate the influence of correspondence distortion, it would be prudent to experimentally examine many acquisition sequences and select one that best reproduces the Seebeck coefficient for the chosen probe arrangement, in comparison with known reference materials, or to combine and average one sequence and its inverse. Furthermore, the thermal gradient heating rate should be reduced in comparison to the acquisition delay. Standard commercial instrumentation feature selectable switching and voltage channel acquisition rates. These rates are inversely proportional to the accuracy of the measured signal but directly proportional to the correspondence distortion. Therefore, the selected rate must optimally manage these competing uncertainties.

## 4. Conclusions

We have employed finite element analysis using COMSOL Multiphysics to develop computational Seebeck coefficient metrology simulations. To demonstrate the usefulness of this approach, we have performed these simulations to quantitatively explore the effect of perturbations to the *V* and *T* correspondence under the quasi-steady-state condition. The results indicate the error that staggered acquisition can introduce in the Seebeck coefficient, in addition to other experimental and instrumentation uncertainties. These errors are strongly dependent on the time delay, the acquisition sequence, and the probe arrangement.

## Figures and Tables

**Table 1:** Seebeck coefficients (*µ*V/K) at 300 K calculated by combining *V* and *T* data representing a 0 s, 0.1 s, 0.5 s, and 1 s time delay for the sequences resulting in the largest distortion of the *V* and *T* correspondence

Sequence		*V*:*T*_2_:*T*_1_			*T*_1_:*T*_2_:*V*		
Δ*t* (s)	0	0.2	0.5	1.0	0.2	0.5	1.0
2-Probe	−110.1	−113.3	−115.4	−119.4	−110.6	−108.6	−105.1
4-Probe	−109.8	−114.2	−119.1	−128.1	−108.5	−104.4	−97.8

## Footnotes

1 Certain commercial software is identified in this paper in order to specify the experimental procedure adequately. Such identification is not intended to imply recommendation or endorsement by the National Institute of Standards and Technology, nor is it intended to imply that the software identified are necessarily the best available for the purpose.
